# Evidence for Anti-Viral Effects of Complete Freund’s Adjuvant in the Mouse Model of Enterovirus Infection

**DOI:** 10.3390/vaccines8030364

**Published:** 2020-07-07

**Authors:** Arunakumar Gangaplara, Chandirasegaran Massilamany, Ninaad Lasrado, David Steffen, Jay Reddy

**Affiliations:** 1 School of Veterinary Medicine and Biomedical Sciences, University of Nebraska-Lincoln, Lincoln, NE 68583, USA; arunakumar.gangaplara@nih.gov (A.G.); mchandirasegaran@gmail.com (C.M.); ninaad@huskers.unl.edu (N.L.); dsteffen1@unl.edu (D.S.); 2 Laboratory of Early Sickle Mortality Prevention, Cellular and Molecular Therapeutics Branch, National Heart, Lung, and Blood Institute, National Institutes of Health, Bethesda, MD 20892, USA; 3 Division of Immuno-Oncology, CRISPR Therapeutics, Cambridge, MA 02139, USA

**Keywords:** enterovirus, adjuvant, CFA, BCG

## Abstract

Group B coxsackieviruses (CVBs) belonging to the genus, *Enterovirus* and contain six serotypes that induce various diseases, whose occurrence may involve the mediation of more than one serotype. We recently identified immunogenic epitopes within coxsackieviruses B3 (CVB3) viral protein 1 that induce anti-viral T cell responses in mouse models of CVB infections. In our investigations to determine the protective responses of the viral epitopes, we unexpectedly noted that animals immunized with complete Freund’s adjuvant (CFA) alone and later challenged with CVB3 were completely protected against myocarditis. Similarly, the pancreatitis-inducing ability of CVB3 was remarkably reduced to only 10% in the CFA group as opposed to 73.3% in the control group that received no CFA. Additionally, no mortalities were noted in the CFA group, whereas 40% of control animals died during the course of 21 days post-infection with CVB3. Taken together, our data suggest that the adjuvant effects of CFA may be sufficient for protection against CVB infections. These observations may provide new insights into our understanding of the occurrence of viral infections.

## 1. Introduction

Enteroviruses belonging to the Picornaviridae family are positive-sense, single-stranded RNA viruses. Based on the currently-adopted method of molecular typing of the viral protein 1 (VP1) nucleotide composition, 13 species of enteroviruses have been identified [1]. Infections caused by four enterovirus species, enterovirus A to D, are the most common that occur in humans, especially infants (children less than 1 year of age) and immune-compromised individuals [1,2]. Enteroviruses induce a wide spectrum of illnesses, such as meningitis, encephalitis, paralysis, myocarditis, and rash/foot and mouth disease [2]. Although enteroviral infections can occur anywhere in the world, recent outbreaks of respiratory illness in the United States highlight their growing importance in human health [3,4,5].

We have been studying the cellular and molecular mechanisms of protective immune responses in mouse models, particularly for coxsackieviruses B3 (CVB3) and B4 (CVB4), which are implicated in the causation of myocarditis/dilated cardiomyopathy and Type I diabetes (TID), respectively [6,7]. We have identified three T cell epitopes within the VP1 of CVB3 namely, VP1 681–700, VP1 721–740, and VP1 771–790. We demonstrated that these epitopes bind major histocompatibility-complex class II molecules with varied affinities [8], that can be used to assess antigen-specific T cell responses in infection or vaccine studies. Traditionally, complete Freund’s adjuvant (CFA) is used to assess the immunogenicity of antigens [9], but it is unclear whether adjuvant effects of CFA alone can offer protection against CVB3 because mycobacterial components in CFA can mediate anti-viral responses [10,11]. In this report, we sought to determine the effect of CFA on CVB3 infection in highly susceptible A/J mice and made an unexpected observation that the animals immunized with CFA were found to be completely protected from both myocarditis and pancreatitis induced with CVB3. 

## 2. Materials and Methods

### 2.1. Mice

Six-to-eight-week old, female A/J mice (H-2^a^) were procured from the Jackson Laboratory (Bar Harbor, ME, USA). Animals were maintained according to the institutional guidelines of the University of Nebraska–Lincoln (UNL), Lincoln, NE, and approval for animal studies was granted by the Institutional Animal Care and Use Committee, UNL (protocol ^#^1904, approved 2 January 2020). Mice infected with CVB3 were monitored closely for clinical signs suggestive of distress. All research staff followed biosafety level 2 guidelines while handling the animals. Animals whose clinical signs persisted, did not eat or drink, and failed to move when touched or prodded physically were immediately euthanized. Euthanasia was performed using a carbon dioxide chamber as recommended by the Panel on Euthanasia, the American Veterinary Medical Association. 

### 2.2. Virus Propagation and Infection

The Nancy strain of CVB3 was procured from the American Type Culture Collection (ATCC, Manassas, VA, USA), and the virus was titrated in Vero cells (ATCC). The adherent Vero cells were grown to 80 to 90% confluence in 75cm^2^ flasks in Eagle’s minimum essential medium (EMEM)/10% fetal bovine serum (FBS) and were later infected with CVB3 with multiplicity of infection 1 in EMEM containing no FBS. After incubation at 37 °C for 1 hour with gentle intermittent rocking, maintenance medium (EMEM/2% FBS) was added. Based on the cytopathic effect of virus during the next 1 to 2 days, supernatants containing virus were harvested. After determining 50% tissue culture infective dose (TCID_50_) values based on the Reed–Muench method, the virus stocks were aliquoted and preserved at −80 °C [12]. To infect mice, virus stock diluted in 1× PBS to contain 50 TCID_50_ in 100 µL was administered intraperitoneally (i.p.). We chose this dose based on titration experiments [12] that allowed us to capture pathological changes in both heart and pancreas over a period of 3 weeks by avoiding acute mortalities that usually occur at relatively higher doses within ~10 days post-infection [13,14]. Animals were monitored closely, cages were changed once in 2 days, and body weights were taken daily until termination. In addition, an alternative food and fluid source, trans gel diet (ClearH2O, Portland, ME, USA), was placed on the cage floor as needed. 

### 2.3. Challenge Studies in Animals Immunized with CFA

To investigate the effects of CFA on CVB3 infection, we used CFA that contained heat-killed *Mycobacterium tuberculosis* (M. tb), paraffin oil and mannose monooleate (Difco Laboratories, Detroit, MI, USA) [8,15]. Groups of animals were immunized subcutaneously with or without CFA emulsion (200 µL; Sigma-Aldrich, St. Louis, MO, USA) containing 5 mg/mL of M. tb to a final concentration of 2.5 mg/mL in 1× PBS as a single dose on day −7 in sternal and inguinal regions [16]. Essentially, 200 μL of CFA emulsion contained 500 µg of M. tb extract. Seven days later (day 0), animals were challenged with CVB3 at 50 TCID_50_/mouse, i.p., and after taking body weights and monitoring for mortalities, experiments were terminated on days 20–21 post-infection and tissues were collected for histology. 

### 2.4. Histology

Hearts and pancreata were fixed in 10% phosphate-buffered formalin and processed to obtain 5 µm thick serial sections, ~50 µm apart. All sections were stained by hematoxylin and eosin (H and E). The analysis was performed by a board-certified pathologist blinded to treatment, and total number of inflammatory foci were obtained as reported previously [12,17]. 

### 2.5. Statistics

Generalized linear mixed models were used to analyze the data pertaining to body weights and survival curves using Proc Glimmix in SAS (Version 9.3, SAS Institute Inc., Cary, NC, USA). Graphs were prepared by GraphPad Prism software Version 8.0 (GraphPad Software, Inc. La Jolla, CA, USA). Barnard’s exact test was used to analyze the histological parameters [18].

## 3. Results and Discussion

**Figure 1:**
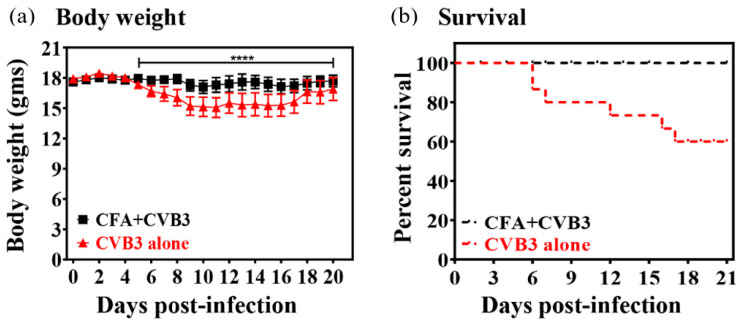
Evaluation of effects of complete Freund’s adjuvant (CFA) in coxsackieviruses B3 (CVB3) infection of A/J mice. (**a**) A/J mice were immunized once (day −7) with or without CFA alone and were challenged with CVB3 on day 0. Body weights were taken up to 20 days post-challenge with CVB3. Mean ± SEM values obtained from two experiments, each involving 5 to 10 mice, are shown (left panel), and (**b**) mortalities noted up to 21 days post-challenge are shown in the survival curves (right panel). **** *p* ≤ 0.0001.

In this report, we provide evidence that immunization with immune-stimulating adjuvants like CFA alone can offer protection against viral infections. In this setting, we used A/J mice that are highly susceptible to CVB infections, with affected animals showing severe pancreatitis and myocarditis within approximately 7 to 10 days post-infection [8,17,19]. Although our primary focus was to determine whether viral peptides can offer protection in challenge studies with CVB3, we made an unexpected observation that the protection offered by CFA emulsions containing viral peptides was indistinguishable (data not shown) from that conferred by immunization with CFA alone. First, we noted that the positive control group (unimmunized) infected with CVB3 showed reduction in body weights by ~20% as expected, whereas none of the animals immunized with CFA alone lost body weight (Figure 1, left panel). Second, mortality patterns were also found to be similar to those of body weights, in that none of the animals immunized with CFA alone died, whereas 40% of animals (6/15) in the control group died (Figure 1, right panel), pointing to the possibility that CFA-immunized animals would also be free of histologic disease.

**Figure 2:**
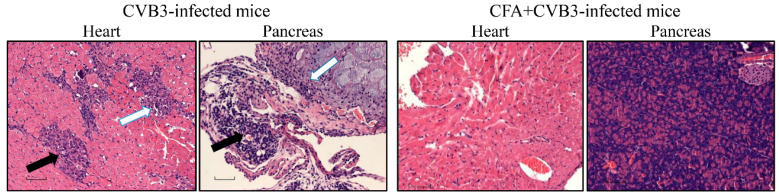
Determination of histological changes in mice immunized with or without CFA and later challenged with CVB3. A/J mice were immunized with or without CFA on day −7 and animals were challenged with CVB3 intraperitoneally (i.p.), on day 0. Hearts and pancreata were examined by hematoxylin and eosin (H and E) staining to determine histological changes. The left panel indicates representative sections from CVB3-infected mice showing multiple inflammatory foci, and necrosis (solid arrow) and mineralization (empty arrow) in the heart, whereas pancreas showed changes such as atrophy and infiltrations (solid arrow) and necrosis and mineralization (empty arrow). The right panel denotes heart and pancreatic sections from CFA+CVB3-infected group, in which lesions were absent. Original magnification: 20×.

To investigate pathological changes, we examined the hearts and pancreata by H and E staining and scored disease severity as we have described previously [12,16,17]. As indicated in Table 1, top panel, and Figure 2, heart sections in 40% (6/15) of the animals from the CVB3-infected group showed myocardial lesions containing inflammatory foci with macrophage infiltrates, necrosis, and mineralization as expected [12,17], but none of the animals in the CFA group had any detectable lesions. Similarly, histological evaluation of pancreatic sections from the CVB3-infected group revealed expected lesions such as atrophy, inflammation, necrosis, and mineralization, whereas in the CFA group, only 10% (1/10) of the animals had detectable lesions (Table 1, bottom panel, and Figure 2). Taken together, the findings that animals immunized with CFA alone were protected against both myocarditis and pancreatitis induced with CVB3 supports the idea that non-specific priming of the immune system with adjuvants like CFA may be sufficient to prevent virus infections. 

Disease protection offered by CFA was rather perplexing to explain, because CFA was not expected to induce antigen-specific immune responses to prevent infection with CVB3. Historically, CFA containing M. tb has been used as a powerful immune-stimulating adjuvant, and it contains immunoreactive molecules, such as N-acetylmuramyl-l-alanyl-d-isoglutamine (muramyl dipeptide) and trehalose 6,6’-dimycolate, all of which promote T helper (Th) 1 cell polarization by inducing interferon (IFN)-γ [10,11,20]. Reports indicate that the Bacillus Calmette–Guérin (BCG) containing mycobacterium can enhance immunogenicity and also promote type I IFN response as shown in various settings such as vaccinations against influenza and hepatitis B viruses in humans [21,22], and infection studies with encephalomyocarditis, murine hepatitis (mouse coronavirus), type 1 and 2 herpes simplex, vaccinia, and foot-and-mouth disease viruses in mice [23,24,25,26,27,28]. Additionally, bacterial CpG nucleotides promote Th1 (IFN-γ) response [29], which is critical for protection against intracellular pathogens and viruses through the production of interleukin-12 by interacting with toll-like receptor-9 [30,31]. Thus, we believe that the disease-protective ability of CFA may reflect non-antigen-specific effects attributable to the adjuvanticity of M. tb. 

However, our data point to a few other possible mechanisms: (i) Exposure to non-specific infections that promote Th1 cytokine responses may offer protection against viral infections possibly by preventing viral replication [10,20,32]. If this holds true, then our data may also potentially provide credence to the hygiene hypothesis [33,34]. (ii) A growing body of evidence suggests that the trained innate memory may be an important property of the innate immune system. Myeloid cells, such as monocytes and macrophages, natural killer (NK) cells, NK-T cells, γδ T cells, and possibly innate lymphoid cells, exposed to a microbe—for example, ‘x’ microbe—can robustly respond to this microbe upon re-exposure, and also for other unrelated microbial stimulations through epigenetic and metabolic reprogramming pathways [35,36,37]. Consistent with this notion, it has been shown that BCG vaccine can offer protection against other unrelated pathogens, such as *Candida albicans, Schistosoma mansoni,* and *Staphylococcus aureus* [38,39,40,41], including autoimmune diseases such as Type I diabetes and multiple sclerosis [42,43,44,45,46]. Likewise, an inverse relationship was observed between BCG vaccination and severity of clinical disease resulting from severe respiratory syndrome coronavirus 2 infection [47,48]. Conversely, it is also possible that exposure to one type of pathogen can suppress immune responses to entirely different types of pathogens. For example, co-administration of oral polio vaccine with BCG at birth can diminish the response to purified protein derivative from BCG [49]. While the BCG vaccine contains live *Mycobacterium bovis* (a pathogen of cattle), CFA contains the killed extract of M. tb (a pathogen of humans). Yet all 13 known mycobacterial species, including the two species identified above, show more than 99% nucleotide similarity, suggesting that all of them may have similar adjuvant properties [50,51]. Furthermore, it has been recently demonstrated that the IFN-γ response induced by BCG can facilitate epigenetic re-programming of hematopoietic stem cells in the bone marrow towards a myeloid lineage that can initiate the central trained immunity in the bone marrow against tuberculosis [52]. A suggestion has been made that the trained monocytes migrating to peripheral tissues that give rise to macrophages can enhance immune responses to incoming/future infections [36]. Since CFA promotes strong IFN-γ response similar to BCG [11], the protective effects of CFA in CVB3 infection could be ascribed to IFN-γ. In support of this proposition, it has been shown that the IFN-γ-deficient mice developed severe CVB3-induced myocarditis suggesting that IFN-γ response plays a critical role in the prevention of CVB infections [53]. Similarly, mice immunized with CFA were also found protected against vesicular stomatitis virus, and CFA-adjuvanted H1N1 influenza vaccine led to enhanced immune response in ferrets [54,55]. Although whether or not it is currently known that trained innate memory is an underlying mechanism for CFA effects as noted in our studies, this aspect may need to be investigated. 

One limitation of our study is that we did not investigate the presence of virus in tissues of CVB3-infected mice. Similarly, it is unknown whether CFA administration can potentiate the production of protective neutralizing antibodies to CVB3, and if so, how long such an effect would last against different doses of virus. Likewise, whether administration of CFA in the face of CVB3 infection can mitigate the disease process is also unknown. At the time of this writing, we could not execute these experiments since our institutional guidelines do not allow any new animal experiments because of the ongoing threat of coronavirus disease-19 pandemic to the public. Nonetheless, our data may provide insights into our understanding of the occurrence of viral infections in the face of pre-existing, non-antigen-specific immune responses generated in response to a potentially wide range of environmental pathogens/microbes or gut microbiota over a period of time, which also may include formation of virtual memory cells [56,57].

## Figures and Tables

**Table 1:** Histological evaluation of hearts and pancreata in mice immunized with or without CFA challenged with CVB3.

Parameters	CVB3 Group	CFA + CVB3-Challenged Group
**Myocarditis**		
**Incidence**	6/15 (40.0)	0/10 (0.0)
**Inflammatory foci**	54.4 ± 17.7	0.0
**Pancreatitis**		
**Incidence**	11/15 (73.3)	1/10 (10.0)
**Atrophy**	7/15 (46.7)	1/10 (10.0)
**Inflammation**	11/15 (73.3)	1/10 (10.0)
**Necrosis**	8/15 (53.3)	0/10 (0.0)
**Mineralization**	4/15 (26.7)	1/10 (10.0)

() indicates percentages; all parameters were significant (*p* ≤ 0.01).

## Abbreviations

BCG	Bacillus Calmette–Guérin
CFA	Complete Freund’s adjuvant
CVB	Coxsackievirus B
EMEM	Eagle’s minimum essential medium
FBS	Fetal bovine serum
IFN	Interferon
I.p.	Intraperitoneal
M.tb	*Mycobacterium tuberculosis*
NK	Natural killer
PBS	Phosphate-buffered saline
RNA	Ribonucleic acid
T1D	Type 1 diabetes
Th	T helper
VP	Viral protein
